# Effects of Commercial Exergames and Conventional Exercises on Improving Executive Functions in Children and Adolescents: Meta-Analysis of Randomized Controlled Trials

**DOI:** 10.2196/42697

**Published:** 2023-10-19

**Authors:** Jinlong Wu, Zhuang Xu, Haowei Liu, Xiaoke Chen, Li Huang, Qiuqiong Shi, Linman Weng, Yemeng Ji, Hao Zeng, Li Peng

**Affiliations:** 1 College of Physical Education Southwest University Chongqing China; 2 Department of Physical Education Tsinghua University Beijing China; 3 Laboratory for Artificial Intelligence in Design Hong Kong China; 4 Faculty of Psychology Southwest University Chongqing China; 5 College of Physical Education Nanchang University Nanchang China

**Keywords:** commercial exergames, exergame, randomized controlled trial, RCT, conventional exercises, executive function, children, adolescent, pediatric, youth, exergaming, randomized, meta-analysis, meta analyses, review method, systematic review

## Abstract

**Background:**

Exergames are promising exercise tools for improving health. To the best of our knowledge, no systematic review has compared the effects of commercial exergames and conventional exercises on improving executive functions (EFs) in children and adolescents.

**Objective:**

This study aimed to investigate the effects of commercial exergames and conventional exercises on improving EFs in children and adolescents.

**Methods:**

Following the PRISMA (Preferred Reporting Items for Systematic Reviews and Meta-Analyses) guidelines, 5 randomized controlled trial (RCT) databases (PubMed, Web of Science, Scopus, PsycINFO, and SPORTDiscus) were searched from their inception to July 7, 2022, to identify relevant RCTs. The Cochrane Collaboration tool was used to evaluate the risk of bias for each study. GRADE (Grading of Recommendations, Assessment, Development, and Evaluation) was used to evaluate the overall quality of evidence.

**Results:**

In total, 8 RCTs including 435 children and adolescents were included in the analysis. Commercial exergames had no significant benefit on overall EFs compared to conventional exercises (Hedges *g*=1.464, 95% CI –0.352 to 3.280; *P*=.06). For core EFs, there was no evidence to suggest that commercial exergames are more beneficial for improving cognitive flexibility (*g*=0.906, 95% CI –0.274 to 2.086; *P*=.13), inhibitory control (*g*=1.323, 95% CI –0.398 to 3.044; *P*=.13), or working memory (*g*=2.420, 95% CI –1.199 to 6.038; *P*=.19) than conventional exercises. We rated the evidence for overall EFs, cognitive flexibility, inhibitory control, and working memory as being of very low quality due to inconsistency (large heterogeneity) and imprecision (low number of people). Additionally, no effects of the intervention were observed in the acute and chronic groups.

**Conclusions:**

We do not have strong evidence to support the benefit of commercial exergaming on EFs because we did not observe a Hedges *g* close to 0 with tight CIs. Further research is needed to confirm this hypothesis.

**Trial Registration:**

PROSPERO CRD42022324111; https://www.crd.york.ac.uk/prospero/display_record.php?RecordID=324111

## Introduction

### Background

Executive functions (EFs) are top-down cognitive processes that control and regulate other cognitive processes while performing intricate cognitive tasks [1]. EFs include 3 core functions, namely, inhibitory control, working memory, and cognitive flexibility [2]. Studies have found that EFs are closely associated with mental health [3,4], academic performance [5], and sleep quality [6] in children and adolescents.

It is well-established that adequate and regular exercise can improve EFs in healthy children and adolescents [7,8]. Exergames (or active video games) are emerging and promising technology-based exercise programs that refer to movement-based interactive video games requiring whole-body exercise [9-11]. Common exergames include Wii Fit, Xbox Kinect, Wii Sports, and Dance Dance Revolution [12]; these exergames platforms and devices comprise a class of commercial exergames [13]. These commercial exergames can increase motivation and engagement from users [14-16] due to their special challenge and interest in games and aim to encourage users to exercise [17]. Commercial exergames have recently become a popular exercise activity with which children and adolescents spend their spare time [18,19]. Previous findings have demonstrated the potential benefits of commercial exergames for both physical health (eg, improving muscle strength [20], balance [21], and cardiopulmonary function [22,23]) and mental health (eg, improving mood states [24], self-esteem [25], and self-efficacy [25]) in children and adolescents.

One systematic review showed that commercial exergames can improve cognitive skills (eg, EFs) in children and adolescents [26]. The main reason that commercial exergames have a positive effect on EFs is that they include several games related to cognitive challenges [26]. However, because the systematic review used passive controls in their comparisons, which hindered the evaluation of the effectiveness of commercial exergames compared to traditional methods, it is still not clear whether exergames offer more advantages for improving EFs than do conventional exercises. Another study performed a meta-analysis to compare the effects of commercial exergames and conventional exercises on the cognitive skills of older adults, and the results did not find that commercial exergames offered better benefits for improving EFs (assessed, for example, using the Stroop task) than conventional exercises [16].

### Objective

To our knowledge, no systematic reviews have compared the effects of commercial exergames and conventional exercises on improving EFs in children and adolescents. This knowledge gap needs to be filled because younger participants have a close affinity for commercial exergames and are also the main beneficiary group [27]. There could be a role for exergames in improving EFs when children (eg, those with neurodevelopmental disorders) are unable or less inclined to engage in conventional exercise. Therefore, this meta-analysis aimed to compare the effects of commercial exergames and conventional physical activity on EFs in children and adolescents.

## Methods

### Design

This meta-analysis was performed in accordance with the PRISMA (Preferred Reporting Items for Systematic Reviews and Meta-Analyses) statement and its accompanying checklist [28] and was registered with PROSPERO (CRD42022324111).

### Study Identification

We searched 5 databases, including PubMed, Web of Science, Scopus, PsycINFO, and SPORTDiscus, for randomized controlled trial (RCT) studies published in English from inception until July 7, 2022, to identify all relevant published articles regarding the effect of exergames on EFs in children and adolescents. The initial search was performed using the following 3 key terms: child/adolescent, exergame, and executive functions. A detailed keyword search strategy can be found in Multimedia Appendix 1. The search keywords for each main term were developed from the search strategies of previous reviews related to commercial exergames, EFs, and children or adolescents [1,29]. Additional literature was identified by searching and reviewing the reference lists of relevant studies.

**Figure 1:**
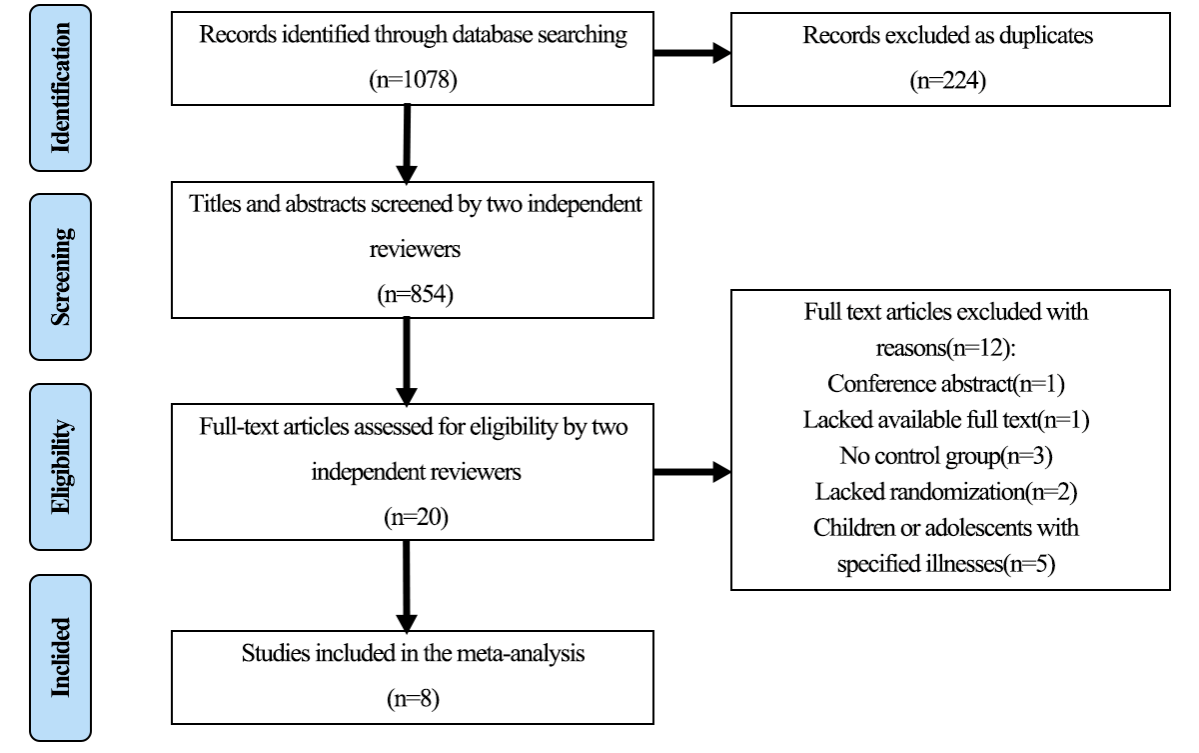
PRISMA (Preferred Reporting Items for Systematic Reviews and Meta-Analyses) study selection diagram.

A total of 1078 records were retrieved from all databases. After removing duplicates and screening titles and abstracts, 854 records were analyzed for eligibility. After full-text screening, 13 articles met the inclusion criteria and were included in this systematic review, and 8 articles were included in the meta-analysis. The article screening process is illustrated in Figure 1.

### Eligibility Criteria

All inclusion criteria followed the PICOS (Participants, Interventions, Comparators, Outcomes, and Study design) framework and included the following criteria: (1) studies that targeted children and adolescents up to 18 years of age [28], (2) the primary intervention was exergames with any modality and the control group underwent conventional exercises, (3) the outcomes involved cognitive performance assessments of overall or core (ie, inhibitory control, cognitive flexibility, and working memory) EFs assessed using questionnaires or computer tasks, and (4) the study design was an RCT published in English in a peer-reviewed journal.

### Study Selection and Data Extraction

Two researchers independently scanned the titles and abstracts, and studies that satisfied the inclusion criteria were retrieved for full-text assessment. Differences between the two researchers were resolved through discussion. If an agreement could not be reached, the final decision was made through discussion with a third researcher. The data were independently extracted by two researchers. The extracted data were related to the document characteristics (first author, publication year, and country or region), participant description (number and age of participants), details of the interventions (exergame group and conventional exercise group), and outcomes measured. If there were multiple control groups in one study, only the data for the control group receiving the conventional exercise were extracted.

### Risk of Bias

We used the revised risk of bias tool described in the Cochrane Handbook version 5.1.0 [30] to categorize the risk of bias of each study, which includes 7 domains, namely, sequence generation, allocation concealment, blinding of assessors, incomplete outcome data, selective outcome reporting, and other sources of bias. The items were rated as having a low, unclear, or high risk of bias. Based on the risk of bias in the individual domains, studies were classified as having a low, unclear, or high risk of bias. Disagreements regarding the risk of bias were resolved by discussion or by consulting a third researcher. The risk of bias for blinding of the outcome assessment was based on the method of outcome assessment (objective or subjective).

### Quality of Evidence

GRADE (Grading of Recommendations, Assessment, Development, and Evaluation) was used to evaluate the quality of evidence [31]. GRADE includes 5 subtraction items, namely, risk of bias, inconsistency, indirect evidence, imprecision, and publication bias. According to the results, the quality of evidence was divided into 4 categories: high, medium, low, and very low. In the GRADE evaluation, there was no evidence of a downgrade to high quality, 1 item was downgraded to medium quality, 2 items were downgraded to low quality, and 3 or more items were downgraded to very low quality. The GRADE assessments were applied independently by two reviewers to judge the certainty of the evidence. If there were disagreements, an experienced researcher made the decision [32].

### Data Analysis

All analyses were implemented using Comprehensive Meta-Analysis version 3 (Biostat). Specifically, when different instruments were used to measure outcome variables, the effect size in each study was calculated using Hedges *g* with 95% CIs between the groups. Hedges *g* was calculated and weighted through inverse variance, thereby accounting for the respective sample sizes, varying outcomes, and cognitive measures [33]. The magnitude of Hedges *g* values was interpreted as trivial (*g*<0.2), small (0.2<*g*<0.5), moderate (0.5<*g*<0.8), and large (*g*>0.8) effect sizes. Heterogeneity across studies was evaluated and graded using the *I*^2^ statistic (very low: *I*^2^<25%; low: 25%<*I*^2^<50%; moderate: 50%<*I*^2^<75%; and high: *I*^2^≥75%) [34]. If *I*^2^≤50%, the research results were considered homogeneous, and a fixed model was used for the meta-analysis. If *I*^2^>50%, then there was heterogeneity among the research results, and a random model was used for the meta-analysis [34]. In addition, the influence of each study on the pooled effect size estimates was examined using a sensitivity analysis. A sensitivity analysis (ie, wherein 1 study was removed) was used to inspect the impact of the retention or removal of outliers and their influence on the overall effect size. As fewer than 10 studies were included in each analysis, publication bias was not investigated. After conducting a meta-analysis for overall EFs, subgroup analyses were performed based on the 3 specific EF domains (inhibitory control, working memory, and cognitive flexibility).

Additional statistical analyses included the following: (1) when studies used ≥2 tests to measure the same variable, the average effect size was calculated; (2) when studies reported ≥2 measurements, only the last measurement was considered; and (3) for studies that reported multiple results on one cognitive task, the result of the more executive demanding condition was included (eg, incongruent trials in the Stroop task) [35].

## Results

### Descriptive Characteristics

Table 1 summarizes the characteristics of the included studies. In total, we analyzed 8 RCT studies [36-42] involving 435 children and adolescents that investigated the difference in effect between commercial exergames and conventional exercises on improving the EFs of healthy children and adolescents. All included studies were published in peer-reviewed English journals. The trials were conducted in the United States (n=4) [37,38,40,41], China (n=3) [39,42,43], and Spain (n=1) [36]. In all studies, the intervention involved the use of commercial exergames to improve EFs. The exergames devices used included Nintendo Wii (n=4) [37,38,42,43], Xbox Kinect (n=3) [36,39,41], and LeapTV console (n=1) [40]. All participants in the control group underwent conventional exercises. There were 2 acute [36,37] and 6 chronic [38-43] interventions. For the 2 acute exergames interventions, the lengths of the single intervention sessions were 15 and 20 minutes. For the chronic exergames intervention, the intervention durations ranged from 4 to 8 weeks, with a frequency between 1 and 5 times per week. The duration of each intervention session ranged from 10 to 30 minutes. Conventional exercises in the control group mainly included conventional exercises [37,40,42,43], running [36,39], and school-as-usual exercises [41].

**Table 1:** Summary of the characteristics of the studies included in the meta-analysis.

Study (country)	Participant description							Exergame group					Control group training			Outcome measured
	Recruited from	Age (years), mean (SD)	Female (%)	Intervention group, n			Platform			Intervention duration						
				Exergame	Control											
Flynn et al [38] (US)	Neighborhoods	13.7 (1.4)	48	70	10	Nintendo Wii			120 min (30 min/session, 1 session/week for 4 weeks)		Exercises			EF^a^: D-KEFS^b^		
Benzing et al [36] (Spain)	Secondary schools	14.5 (1.1)	0	21	23	Xbox Kinect			15 min		Running			EF: D-KEFS		
Flynn and Richert [37] (US)	NR^c^	Range: 6.8-12.9	48	35	36	Nintendo Wii			20 min		CE^d^			IC^e^: Flanker task		
Xiong et al [42] (China)	Childcare center	Range: 4-5	50	30	30	Nintendo Wii			800 min (20 min/session, 5 sessions/week for 8 weeks)		CE			CF^f^: DCCS^g^		
Gao et al [40] (US)	Neighborhoods	4.7 (0.7)	59	18	14	LeapTV console			1800 min (30 min/session, 5 sessions/week for 12 weeks)		CE			CF: DCCS		
Gai et al [39] (China)	Kindergartens	5.7 (0.5)	NR	32	28	Xbox Kinect			360 min (20 min/session, 3 sessions/week for 6 weeks)		Running			WM^h^: Backward digit; IC: Flanker task, CF: DCCS		
Layne et al [41] (US)	Primary school	Range: 8-9	23	19	21	Xbox Kinect			200 min (10 min/session over 4 weeks)		School-as-usual exercises			IC: Go/No-Go		
Liu et al [43] (China)	Preschools	4.9 (0.3)	52	24	24	Nintendo Wii			600 min (30 min/session for 20 sessions over 4 weeks)		CE			IC: Go/No-Go; CF: DCCS; WM: “Mr. Ant”		

^a^EF: executive function.

^b^D-KEFS: Delis-Kapan executive function system.

^c^NR: not reported.

^d^CE: conventional exercise.

^e^IC: inhibitory control.

^f^CF: cognitive flexibility.

^g^DCCS: dimensional change card sort.

^h^WM: working memory.

### Methodological Quality

**Figure 2:**
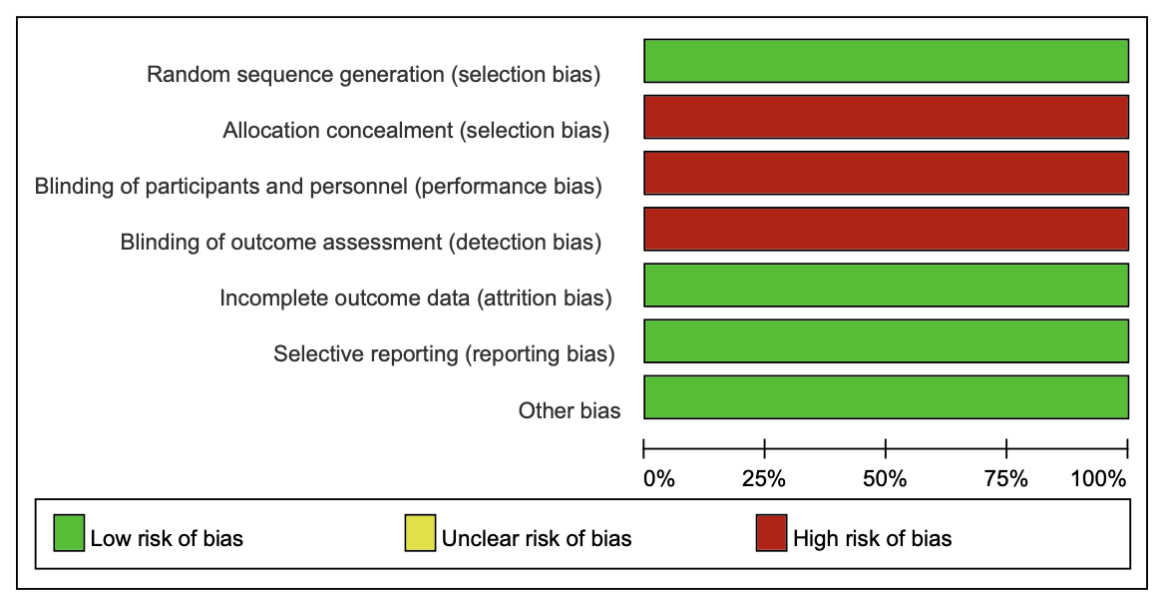
Risk of bias graph. Percentages represent the risk of bias.

All the included studies had a low risk of bias in random sequence generation, incomplete outcome data, selective reporting, and other biases. We awarded a high risk of bias for allocation concealment and blinding. Allocation concealment and blinding are difficult in many exercise intervention trials (Figure 2). The overall quality of evidence according to the GRADE approach is presented in Table 2. We rated the evidence for overall EFs, cognitive flexibility, inhibitory control, and working memory as very low quality due to inconsistency (large heterogeneity) and imprecision (low number of people). Details of the GRADE criteria are provided in Multimedia Appendix 2.

**Table 2:** Summary of GRADE (Grading of Recommendations, Assessment, Development, and Evaluation) assessment of the effect of exergames on executive functions (EFs).

EF	Certainty assessment						Participants, n		Effect, absolute SMD^a^ (95% CI)	Certainty
	Studies^b^, n	Risk of bias	Inconsistency	Indirectness	Imprecision	Other considerations	Experimental	Control		
Overall EF	4	Not serious	Serious^c^	Not serious	Serious^d^	None	147	85	1.464 (–0.352 to 3.280)	Very low
CF^e^	5	Not serious	Serious^c^	Not serious	Serious^d^	None	125	119	0.906 (–0.274 to 2.086)	Very low
IC^f^	3	Not serious	Serious^c^	Not serious	Serious^d^	None	77	75	1.323 (–0.398, 3.044r)	Very low
WM^g^	2	Not serious	Serious^c^	Not serious	Serious^d^	None	56	52	2.420 (–1.199 to 6.038r)	Very low

^a^SMD: standardized mean difference.

^b^All studies were randomized controlled trials.

^c^Large heterogeneity was observed among the included studies (*I*^2^ >75%).

^d^The overall number of individuals included in the trials was low (<400 individuals in both treatment groups).

^e^CF: cognitive flexibility.

^f^IC: inhibitory control.

^g^WM: working memory.

### Meta-Analysis of Effects on Overall and Core EFs

**Figure 3:**
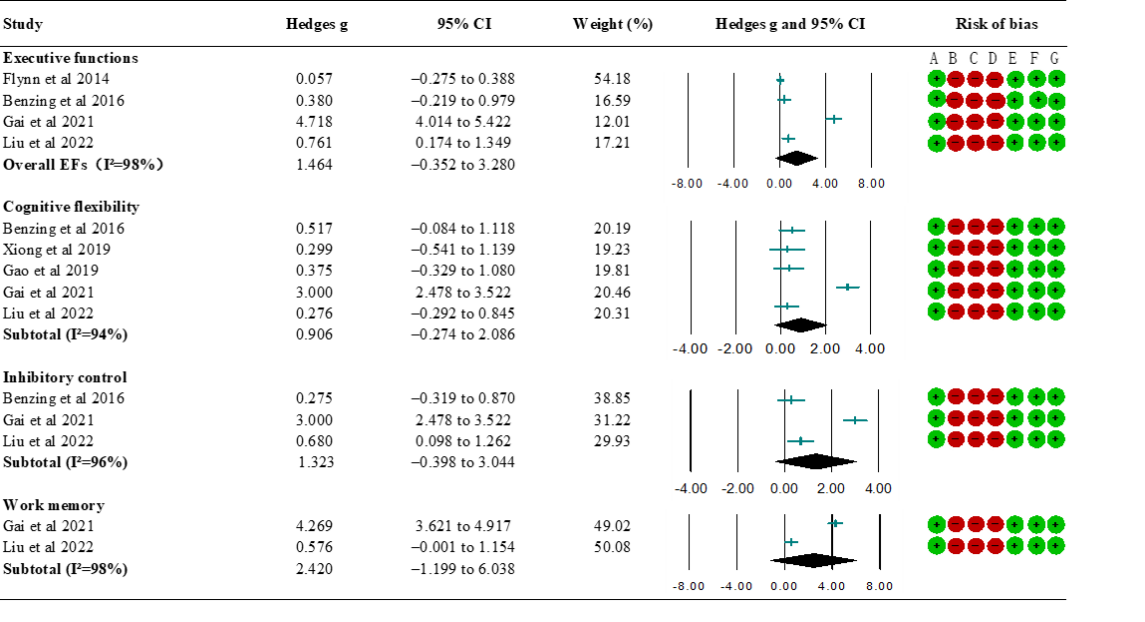
Forest plot for the meta-analysis of the effect of commercial exergames and conventional exercises on improving EFs. EF: executive function.

The results of the meta-analysis are shown in Figure 3 [36,38-40,42,43]. Of the 8 studies included, 4 [36,37,39,43] examined the effects of commercial exergames on overall and core EFs. The EF tasks from the 2 included studies were integrated into 3 core EF domains. We used the random effects model for all comparisons because of the high heterogeneity among the included studies. Specifically, the pooled Hedges *g* for overall EFs was 1.464 (95% CI –0.352 to 3.280; *P*=.06), with large heterogeneity (*I*^2^=76%). The subgroup results showed a nonsignificant effect size on cognitive flexibility in 5 studies [36,39,40,42,43] (*g*=0.906, 95% CI –0.274 to 2.086; *P*=.13), inhibitory control in 3 studies [36,39,43] (*g*=1.323, 95% CI –0.398 to 3.044; *P*=.13), and working memory in 2 studies [39,43] (*g*=2.420, 95% CI –1.199 to 6.038; *P*=.19), with large heterogeneity (*I*^2^=94%, 96%, and 98%, respectively). Sensitivity analyses were carried out for overall EFs and cognitive flexibility, and 1 study [39] was found to be an outlier (*z*=11.272 and 13.141, respectively); thus, a “one study removed” test was performed. The single effect size score specified a change of –0.533 and –0.874, respectively, but was significant (*P*=.11 and *P*=.15, respectively) and within the 95% CI.

To further analyze the effect of exergames on overall and core EFs between acute and chronic interventions, we calculated the effect size for these 2 types of interventions (Table 3). The results showed that these findings were not statistically significant among acute and chronic interventions. Notably, the results have high heterogeneity and wide CIs, which can downgrade the consistency and precision of the evidence.

**Table 3:** Summary of acute and chronic subgroup analyses.

Subgroup		Studies, n	Hedges *g* (95% CI)	*I*^2^ (%)	*P* value
**Executive functions**					
	Acute intervention	2	0132 (–0.155 to 0.420)	0	.36
	Chronic intervention	2	2.713 (–1.147 to 6.574)	98.602	.17
**Cognitive flexibility**					
	Chronic intervention	4	0.989 (–0.488 to 2.465)	95.483	.19
**Inhibitory control**					
	Chronic intervention	2	1.828 (–0.438 to 4.094)	97.047	.11

## Discussion

### Principal Findings

The current review investigated the effects of comparing commercial exergames and conventional exercises on improving EFs in children and adolescents based on the outcome data of 8 studies. However, there is a lack of evidence supporting the benefit of commercial exergaming on EFs because of the very high heterogeneity and wide CIs in our results.

To some extent, our results are consistent with those of previous studies. Sala et al [44] performed a reanalysis of a recent meta-analysis in which Stanmore et al [45] claimed that exergames exert a positive effect on cognition, explained the impact of exergames on cognition as small or null, and found no evidence that exergames improve cognitive ability. Notably, the study population was not restricted. Another 2 studies with meta-analyses of RTCs examined the effectiveness of exergames in improving EFs for older people and found that exergames were not superior to conventional exercises in improving EFs among older adults [46,47]. Our findings are consistent with these reviews.

Soares et al [16] conducted a meta-analysis and explained that the difference between exergames and conventional exercises may be related to the intervention of the control group (conventional exercises) with regard to cognitive demand. Specifically, exergames appear to be more effective for global cognitive performance than conventional exercises with low cognitive demand, in contrast with no advantages in improving cognitive performance when comparing exergames versus conventional exercises with higher cognitive demand. Another study [48] compared virtual reality–based exercises with high versus low cognitive demand and found that exercises with high cognitive demand were more beneficial for EFs than ones with low cognitive demand. Although some studies found that cognitive training with exercise can improve cognitive performance [49,50], it is noteworthy that we only included studies using commercial exergames as the intervention; most commercial exergames platforms and devices (eg, Xbox Kinect and Wii Sports) were not designed to improve one particular cognitive function. Commercial exergames have fewer cognitive components than do professional cognitive training programs. This might be one reason why there was no significant difference between commercial exergames and conventional exercises in improving EFs in children and adolescents in our study.

Another reason may be related to the subjects included in our study. Our study only included healthy children and adolescents rather than children and adolescents with EF impairments (eg, autism spectrum disorder and attention-deficit/hyperactivity disorder). The intervention duration was only 4 to 8 weeks, and based on commercial exergames with low cognitive demands, healthy children and adolescents may require a longer intervention duration to achieve the expected improvement [51]. When the time available for the intervention is limited, healthy children and adolescents may receive the same benefits from commercial exergames and conventional exercises. This may be another reason for the lack of significant differences.

Although these results were not consistent with our expectations, we believe that they are highly encouraging. Given their benefits and advantages (eg, emotional experiences, high feasibility, and usability), exergames can attract children and adolescents to gameplay, keep them physically active, and may play an important role in improving cognitive functions in children or adolescents who are unable or less inclined to engage in conventional exercise. Engaging in exergames is considered a more active lifestyle; thus, we should encourage the development and design of exergame platforms, particularly customized exergames, that is, exergame interventions or platforms designed based on the training or rehabilitation aims of different populations. Studies are needed to examine whether the customization of exergames can help target populations obtain more benefits in cognitive performance.

### Limitations

This study had several limitations. First, only 8 RTCs reporting the effectiveness of exergames compared with conventional exercise in improving EFs were included in this study, which may have impacted the precision and variability of the estimates. Second, there was large heterogeneity between the included studies in this meta-analysis, the source of which was not found due to data limitations. These heterogeneities might be related to intervention programs, exergame platforms, or other aspects of the included studies, which may have reduced the quality of evidence and negatively impacted the precision and variability of the estimates.

Finally, because only a limited number of studies were included in this review, we could not identify potential moderators (eg, age). This suggests that more work is needed in the field to further examine and confirm the findings of this study.

### Conclusions

There is a lack of evidence of the benefits or harms of commercial exergaming because we did not find a Hedges *g* close to 0 with tight CIs. Further research is needed to confirm this hypothesis.

## Appendix

Multimedia Appendix 1 Detailed search strategy.

Multimedia Appendix 2 Details of GRADE Criteria.

## Abbreviations

EF: executive function
GRADE: Grading of Recommendations, Assessment, Development, and Evaluation
PICOS: Participants, Interventions, Comparators, Outcomes, and Study design
PRISMA: Preferred Reporting Items for Systematic Reviews and Meta-Analyses
RCT: randomized controlled trial
