# Correction: Effects of Commercial Exergames and Conventional Exercises on Improving Executive Functions in Children and Adolescents: Meta-Analysis of Randomized Controlled Trials

**DOI:** 10.2196/55167

**Published:** 2023-12-28

**Authors:** Jinlong Wu, Zhuang Xu, Haowei Liu, Xiaoke Chen, Li Huang, Qiuqiong Shi, Linman Weng, Yemeng Ji, Hao Zeng, Li Peng

**Affiliations:** 1 College of Physical Education Southwest University Chongqing China; 2 Department of Physical Education Tsinghua University Beijing China; 3 Laboratory for Artificial Intelligence in Design Hong Kong China; 4 Faculty of Psychology Southwest University Chongqing China; 5 College of Physical Education Nanchang University Nanchang China

In “Effects of Commercial Exergames and Conventional Exercises on Improving Executive Functions in Children and Adolescents: Meta-Analysis of Randomized Controlled Trials” (JMIR Serious Games 2023;11:e42697) the authors made one correction.

In the corrected article a footnote (*) to denote equal contributions for authors Jinlong Wu and Zhuang Xu was added as follows:

Jinlong Wu 1^*^, MA;  Zhuang Xu 1^*^, MA;  Haowei Liu 1, MA;  Xiaoke Chen 2, MA;  Li Huang 1, MA;  Qiuqiong Shi 3, PhD;  Linman Weng 4, MA;  Yemeng Ji 1, MA;  Hao Zeng 5, MA;  Li Peng 1, PhD

 *these authors contributed equally

The equal contributions footnote did not appear in the originally published article.

The correction will appear in the online version of the paper on the JMIR Publications website on December 28th 2023,  together with the publication of this correction notice. Because this was made after submission to PubMed, PubMed Central, and other full-text repositories, the corrected article has also been resubmitted to those repositories.

