# Supplementary material for: Effects of Commercial Exergames and Conventional Exercises on Improving Executive Functions in Children and Adolescents: Meta-Analysis of Randomized Controlled Trials
Source: JMIR Serious Games. 2023 Oct 19;11:e42697. doi: 10.2196/42697 (PMC10623224; doi:10.2196/42697)
Supplement: Multimedia Appendix 1 [file games_v11i1e42697_app1.docx]

Detailed of Search Strategy

| Database | Search strategy |
| --- | --- |
| Pubmed: 102  Web of science: 417  Scopus: 504  PsyclNFO: 11  Sport：44 | children OR childhood OR school-age OR youth OR adolescents OR teenagers OR students  “executive function” OR “executive dysfunction” OR “working memory” OR “problem-solving” OR “decision making” OR planning OR “inhibitory control” OR “ response inhibition” OR inhibit* OR shifting OR switching OR neuropsychologic* OR “cognitive control” OR set-shifting OR “cognitive flexibility” OR “impulse control” OR attention OR cogniti* OR “mental flexibility” OR “mental set shifting”OR self-control OR “ behavioral inhibition” OR “ interference control” OR reasoning  exergam* OR “active video gam*” OR “exercise gam*” OR Wii OR Xbox OR X-box OR Kinect OR Nintendo OR “ Balance board” |
|  |  |
|  |  |
