# Supplementary material for: Effects of Commercial Exergames and Conventional Exercises on Improving Executive Functions in Children and Adolescents: Meta-Analysis of Randomized Controlled Trials
Source: JMIR Serious Games. 2023 Oct 19;11:e42697. doi: 10.2196/42697 (PMC10623224; doi:10.2196/42697)
Supplement: Multimedia Appendix 2 [file games_v11i1e42697_app2.docx]

Detailed of GRADE Criteria.

| Downgrade | Risk of Bias | Inconsistency | Indirectness | Imprecision | Publication Bias |
| --- | --- | --- | --- | --- | --- |
| −1 | If 1 or more of the 3 criteria (randomization, masking, dropout rate ≤30%) is not met in 10–30% of trials included in the systematic review | I2 50–74% | The question being addressed by the guideline panel is different from the available evidence regarding the PICO or regarding the characteristics of those who will deliver the intervention | (a) The overall number of individuals included in trials is low (less than 400 individuals, both treatment arms) OR (b) the 95% confidence interval includes both 1) no effect and 2) appreciable benefit (RR: ≤0.75) or appreciable harm (RR: ≥1.25)[∗](https://www.sciencedirect.com/science/article/pii/S1525861020307374#tblS2fnlowast) | - |
| −2 | If 1 or more of the 3 criteria (randomization, masking, dropout rate ≥30%) is not met in >30% of trials included in the systematic review | I2 ≥ 75% | The question being addressed by the guideline panel is markedly different from the available evidence regarding the PICO or regarding the characteristics of those who will deliver the intervention | (a) the overall number of individuals included in trials is very low (fewer than 400 individuals, both treatment arms) AND (b) the 95% confidence interval includes both 1) no effect and 2) appreciable benefit (RR: ≤0.75) or appreciable harm (RR: ≥1.25)[∗](https://www.sciencedirect.com/science/article/pii/S1525861020307374#tblS2fnlowast) | Egger's test (P value) <. |

NOTE: PICO, Population, Intervention, Comparison, and Outcomes. For dichotomous outcomes, “*no effect*” means an estimate with a confidence interval that crosses 1; appreciable benefit or appreciable harm means that the upper or lower confidence limit crosses a risk of 1.25 or 0.75. For continuous outcomes, “no effect” means an SMD with a confidence interval that crosses zero; appreciable benefit or appreciable harm means that the upper or lower confidence limit crosses an effect size of 0.5 in either direction.

The table of GRADE criteria was cited from Demurtas, J. , et al 2020. (Physical Activity and Exercise in Mild Cognitive Impairment and Dementia: An Umbrella Review of Intervention and Observational Studies)
